# Novel deletion alleles of a *C. elegans* gene C38D4.9, named as *tm4476* and *tm4561*

**DOI:** 10.17912/W2HH32

**Published:** 2017-10-03

**Authors:** Sawako Yoshina, Sayaka Hori, Yuji Suehiro, Shohei Mitani

**Affiliations:** 1 Department of physiology, School of medicine, Tokyo women’s medical university, Shinjuku-ku, Tokyo, 162-8666, Japan

**Figure 1.**
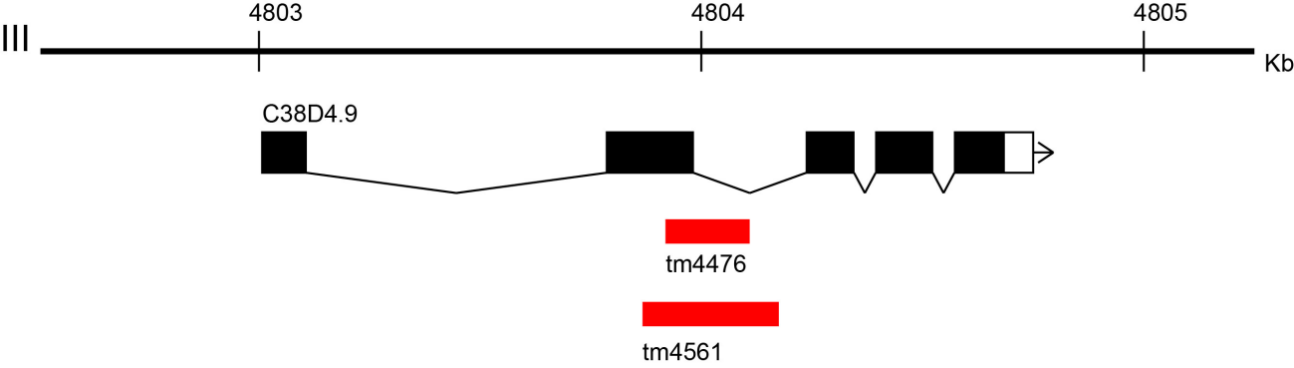
**Figure 1.** Location of the novel alleles

## Description

We report *tm4476* and *tm4561* as novel alleles of *C38D4.9* gene that is an ortholog of human METTL5 (methyltransferase like 5)1. Human METTL5 function has not been reported. The alleles were isolated from the comprehensive screening of gene deletions generated by TMP/UV2. In the screening, both the alleles were detected by nested PCR using the following primer sets, 5’-CCGCCTATATCATGGCGCTT-3’ and 5’-GCTGGTAGTTGCCACCGCAT-3’ for first round PCR and 5’-ACTATCGAAGCCGCGCTTCA-3’ and 5’-AACCCCAACTCCGTCCCATT-3’ for second round PCR. By Sanger sequencing, the 30 bp flanking sequences of the alleles *tm4476* and *tm4561* were identified as GTATGAGTTGGAGACTGTTTTAGGAGTTGA-(191 bp deletion)-GTACTCTTTAAAAGCGCACATCTTTCTGAA and TAATTGATATCGGATGTGGATGTGGAATGT-(309 bp deletion)-ATTTTCGGCTAAAAATCGTATTTGGGTAAT, respectively (Fig. 1). Based on the information about the splicing isoforms of *C38D4.9* (WormBase3 WS259), the second exon of *C38D4.9* is deleted in *tm4476* and *tm4561*.

## Reagents

FX04476 *C38D4.9(tm4476)* III (Not outcrossed)

FX04561 *C38D4.9(**tm4561**)* III (Not outcrossed)
